# In vitro analysis of mechanism of pulsed-laser thrombolysis

**DOI:** 10.1371/journal.pone.0262991

**Published:** 2022-01-27

**Authors:** Daisuke Yamashita, Yuji Matsumoto, Yoshinori Tamaoki, Yukio Ueda, Hiroyuki Okada, Toshiyuki Kawashima, Yutaka Yamashita, Teiji Nakayama, Kazuo Umemura

**Affiliations:** 1 Central Research Laboratory, Hamamatsu Photonics K.K., Hamamatsu City, Shizuoka-Pref., Japan; 2 Department of Pharmacology, Hamamatsu University School of Medicine, Hamamatsu City, Shizuoka-Pref., Japan; 3 Global Strategic Challenge Center, Hamamatsu Photonics K.K., Hamamatsu City, Shizuoka-Pref., Japan; 4 Department of Neurosurgery Hamamatsu Medical Center, Hamamatsu City, Shizuoka-Pref., Japan; Sun Yat-Sen University, CHINA

## Abstract

Thrombolytic therapy in the treatment of cardiogenic acute cerebral embolism caused by coagulated blood carries the risk of hemorrhagic complications, and there is a need to develop safer and more reliable treatment methods. Laser thrombolysis therapy, which utilizes the difference in energy absorption between the thrombus and the arterial wall, has shown promise as a new treatment method because it can selectively act only on the thrombus. It has not been applied clinically, however, and one of the main reasons for this is that its underlying mechanism has not been elucidated. We developed a pulse laser thrombolysis system for treating cerebral blood vessels that consists of a diode-pumped solid-state neodymium-yttrium aluminum garnet laser, which has excellent stability and maintainability and is suitable for clinical applications coupled to a small-diameter optical fiber. Moreover, we analyzed the mechanisms that occur during pulsed laser irradiation of transparent glass tubes and gelatin phantoms. We found that bubbles form as a thermal effect in addition to ablation of the pulsed laser irradiation. Furthermore, we detected no shock waves or water jets associated with the bubbles. We analyzed the bubbles’ dynamics and growth rate, and their effect on a rabbit blood clot phantom. We concluded that the bubbles generated by the laser irradiation physically cut the thrombus and thereby had a thrombectomy effect. We believe that this study will clarify the mechanism of laser thrombolysis therapy and contribute greatly to the realization of its clinical application.

## Introduction

According to a report by the Ministry of Health, Labour and Welfare, cerebrovascular disease accounts for one in ten deaths in Japan. In particular, cardiogenic acute cerebral embolism, in which blood flow to the brain is suddenly interrupted, often causes serious, if not fatal, symptoms [1, 2]. For this reason, blood flow during cerebral embolism needs to be restarted before the brain tissue is irreversibly damaged (within approximately 4.5 hours), and thrombolytic therapy with drugs (tissue plasminogen activator: tPA) is a first choice.

On the other hand, thrombolytic therapy increases the risk of hemorrhagic complications, so safer and more reliable treatment methods need to be developed [3–7]. There have been rapid advances in endovascular interventional techniques, such as embolectomy with mechanical embolus removal using the cerebral ischemia (Merci) retrieval system (Concentric Medical, Mountain View, CA, USA) and mechanical clot aspiration with the Penumbra system (Penumbra, Alameda, CA, USA). However, endovascular interventional techniques have clinical issues, such as the risk of vascular injury.

At the same time, selective laser thrombolysis, which utilizes the difference in energy absorption between the thrombus and the arterial wall in the 300–600 nm band, has been attempted, and studies using a 577 nm dye laser and a 308 nm excimer laser have been reported [8–15]. Notably, the difference in the range from 500 to 600 nm is expected to have a selective effect on fibrin and redblood-cell-rich red thrombi, which cause cardiogenic cerebral embolism [10]. For instance, Viator et al. reported laser thrombolysis using a pulsed laser with a wavelength of 532 nm and a pulse width of 50–200 μs [16]. They reported that the irradiation generated air bubbles and suggested that bursting bubbles caused a water jet effect that helped remove the thrombus, although they did not clarify the underlying removal mechanism. Laser thrombolysis, like any treatment in cerebral vasculature, involves a certain amount of risk and it has not been used clinically even though many researchers have vigorously studied the effects of laser thrombolysis therapy. This may be due in large part to the fact the mechanism underlying thrombus removal has not yet been elucidated.

We are developing a diode-pumped all-solid-state neodymium-yttrium aluminum garnet (Nd:YAG) laser system (wavelength: 532 nm, pulse width: 50–200 µs, repetition: 1–10 Hz) for clinical applications. We believe that a fully solid-state laser is suitable because of its ease of maintenance and stability. To enable irradiation of cerebral blood vessels, our system consists of a low-peak-power pulse laser with and an optical fiber that has a core diameter of 100 µm. Our device is easily portable and can be used in in vivo experiments. In this study reported here, clear transmission images were obtained of transparent glass tubes and gelatin phantoms, and a detailed analysis of the mechanism of thrombus removal was attempted in an experiment on a rabbit blood clot phantom. We found that bubbles were generated as a thermal effect of the pulsed laser irradiation. In particular, the growth rate of the bubbles did not exceed the acoustic velocity, and there was no shock wave or water jet as had been suggested in previous reports. Hence, we concluded that physical cutting by the bubbles themselves the thrombus is removed in therapy. We believe that the results of this study will be an important contribution to the realization of the clinical application of laser thrombolysis therapy.

## Materials and methods

### Laser system

**Fig 1:**
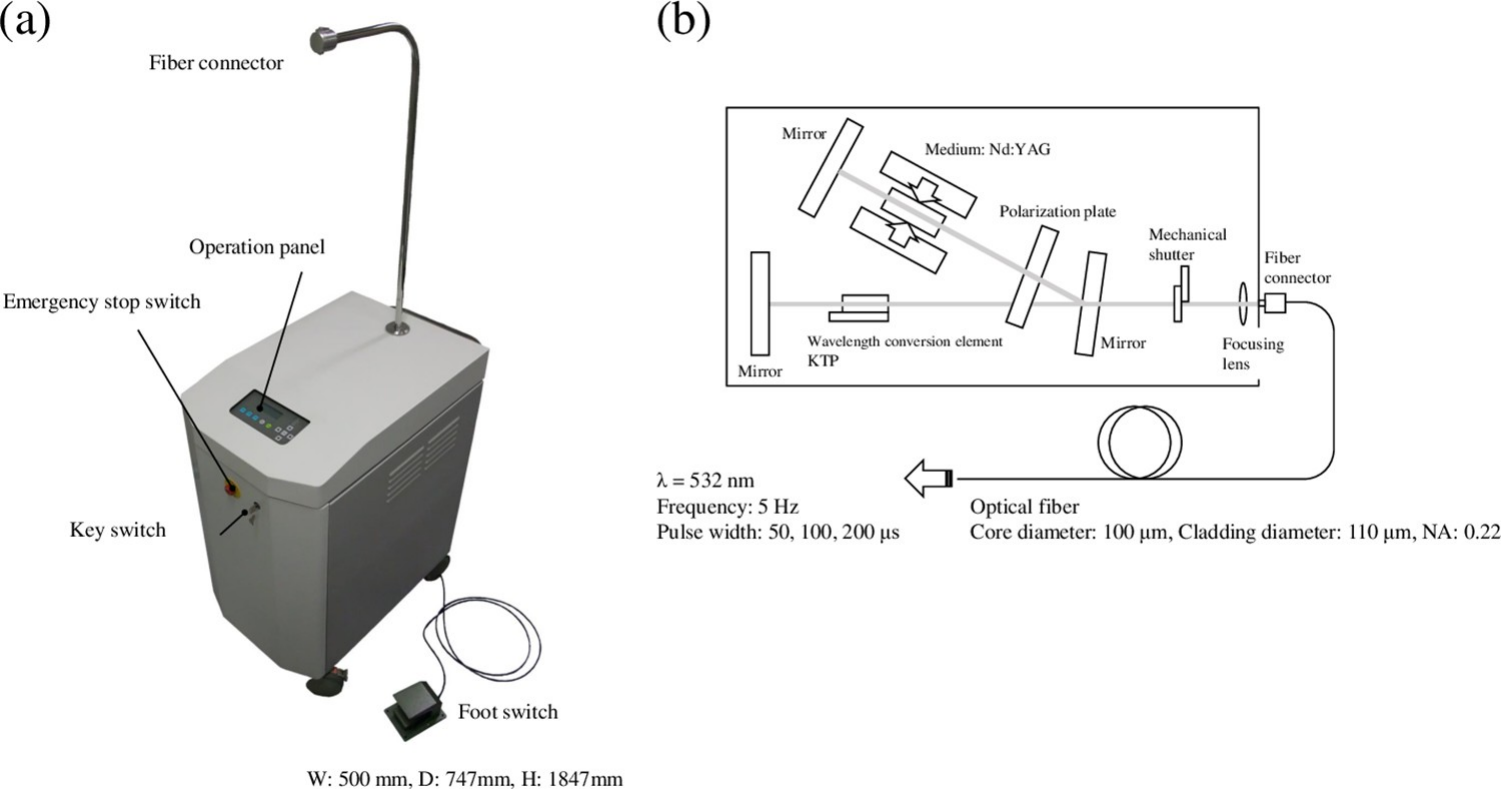
Laser system: LA1292. (a) External view and (b) block diagram. The transportable laser irradiation system for laser thrombolysis therapy with a doddle enclosure for preventing light leakage. Width: 500 mm, Depth: 747 mm, Height: 1847 mm (including the arm).

A prototype laser system (LA1292; Hamamatsu Photonics, Shizuoka, Japan) was developed as a pulse laser source for laser thrombolysis with a double enclosure structure for the purpose of preventing light leakage. A block diagram and a photo of the system are shown in Fig 1, and its specifications are listed in Table 1. This system has a diode-pumped second-harmonic all-solid-state Nd:YAG laser (wavelength: 532 nm). Since the laser system has a relatively broad pulse width in the microsecond range, a second harmonic can be efficiently obtained by placing a potassium titanyl phosphate (KTP: KTiOPO_4_) crystal inside the resonator. A pulse laser was coupled into an optical fiber by a collimating lens installed in the cabinet. To treat the thrombus at the middle cerebral artery (MCA) M2 area (vessel diameter ≤ 1 mm), a small-diameter quartz fiber (F-MCB-T; Newport Corporation, Irvine, CA, USA) was used (core diameter: 100 µm, cladding diameter: 110 µm, numerical aperture (NA): 0.22). Thus, the laser system has more than 100 mW in average power at the tip of the fiber, with a pulse width of 50, 100, or 200 µs and a repetition rate of 1 to 10 Hz. The connector for the optical fiber was placed at the apex of the arm at a sufficient height to accommodate the movements of a person during surgery. The connector is a non-contact type that uses focusing optical system to prevent damage during replacement of the fiber. The pulsed laser irradiation is controlled by a foot switch that is linked to a mechanical shutter installed in the enclosure, allowing the surgeon to keep both hands-free during laser irradiation.

**Table 1:** Specifications of LA1292.

Wavelength	532 nm
Pulse Width	50, 100, 200 μs (fixed value)
Frequency	1–10 Hz (variable)
Average Power	Max 120 mW

Second-harmonic all-solid-state Nd:YAG laser system (wavelength: 532 nm). The repetition rate can be varied in the range of 1–10 Hz in 1 Hz increments. The maximum average power is 120 mW (pulse width: 100 μs, repetition: 5 Hz).

### Dynamic analysis of laser irradiation

**Fig 2:**
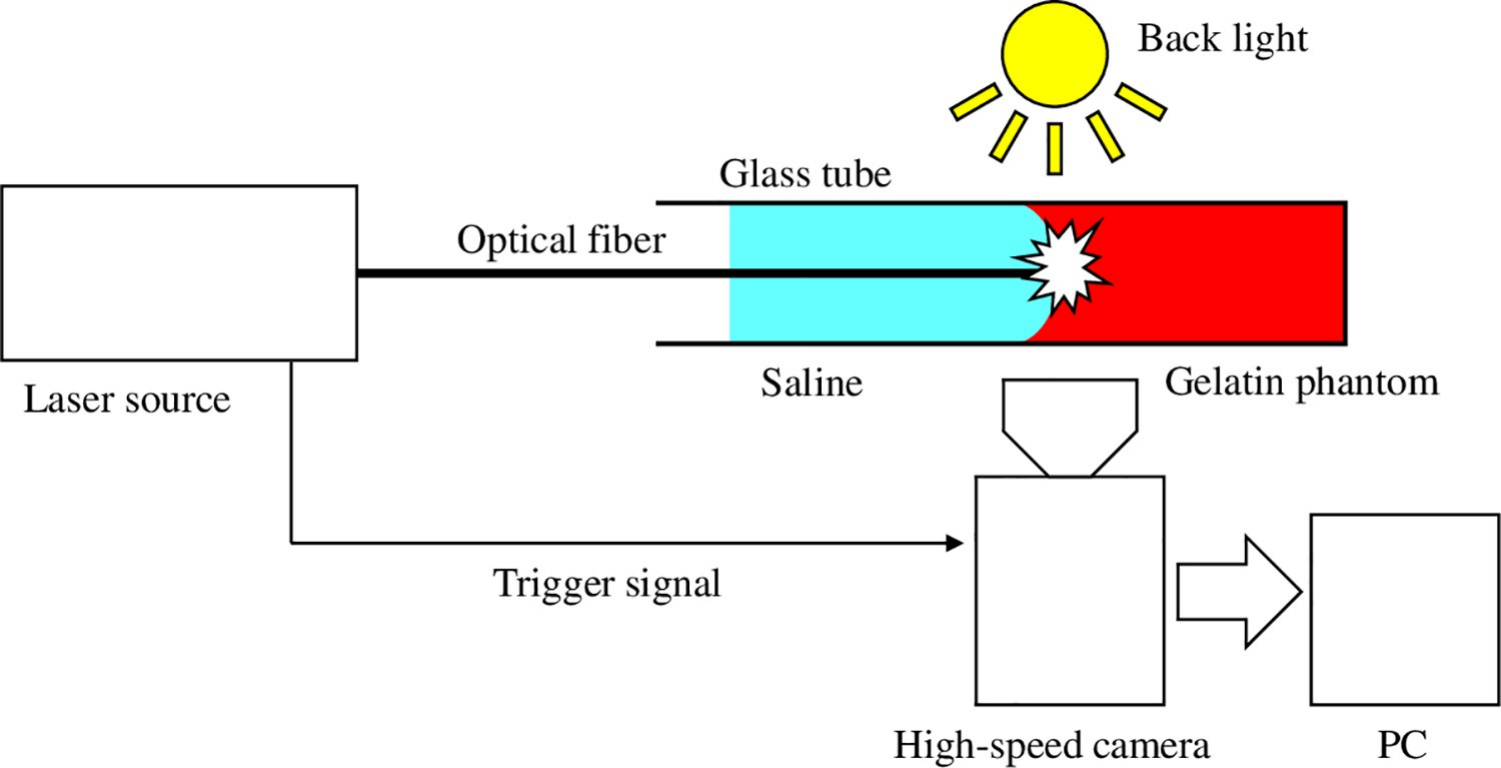
Block diagram of the in vitro experiment using a high-speed camera for dynamic analysis of the pulsed laser irradiation reaction.

A high-speed camera (FASTCAM SAPHC; Photoron, Tokyo, Japan) was used to analyze the single pulse reaction. A block diagram of the experimental setup is shown in Fig 2. We used gelatin phantoms encapsulated in glass tubes to obtain detailed transmission images. A dye (Direct Red 81; Sigma-Aldrich, St. Louis, MO, USA) was added to 10 wt% gelatin (G2500; Sigma-Aldrich, St Louis, MO, USA) to make the phantom’s absorbance at 532 nm equal to that of blood [15, 17]. The hardness of gelatin depends on its protein concentration, but it is difficult to obtain a correlation between the mechanical strength of a gelatin phantom and that of an actual thrombus [11]. Therefore, we determined the concentration of gelatin on the basis of animal studies and experience. A glass tube with inner and outer diameters of 2 mm and 3 mm (FPT-300; Fujirika, Osaka, Japan) was filled with a 10 wt% aqueous solution of gelatin, cooled until the gelatin coagulated, and then filled with saline. An optical fiber was inserted into the glass tube, and pulsed laser irradiation was performed. The surface of the gelatin phantom was bent into a U-shape along the glass wall due to surface tension. Therefore, the positions of the fiber tip and the surface of the gelatin phantom were judged visually using the camera image, and the distance between the tip of the fiber and the surface of the gelatin phantom was controlled within a micrometer of 0.5 mm. The camera frame rate and the exposure time were set at 2 µs/f and 10 µs, respectively. The amount of incident energy was 4–16 mJ/pulse (N = 5 in each irradiation condition). Bubble size was calculated from the measured length of major and minor axes at the maximum size, considering the bubble’s cross section to be elliptical. To prevent heat dissolution of gelatin before irradiation, room temperature during the experiment was set at 25℃.

### Evaluating the size of debris

Male New Zealand White (NZW) rabbits (11 to 12 weeks old) purchased from Japan SLC (Shizuoka, Japan) were used in the present study. All surgery was performed under isoflurane inhalation anesthesia (Pfizer, New York, NY, USA) under strictly standardized conditions. All animal experiments were approved by the animal care and use committee of the Hamamatsu University School of Medicine (No. 2017025).

The size of debris generated by irradiation was evaluated using a rabbit blood clot phantom. Whole blood was collected from the carotid artery of NZW rabbits under isoflurane anesthesia. After the blood was collected, the rabbits were sacrificed with an overdose of pentobarbital. Blood clots in a polyethylene tube were made by incubation for 40 to 50 min. at 37℃. As with the gelatin phantom, a glass tube with an inner diameter of 2.0 mm was filled with clot phantom along with saline. Average power was 0, 20, 40, 60, and 80 mW, pulse width was 100 μs, frequency was 5 Hz, and irradiation time was 10 s (0 mW: N = 3, 20–80 mW: N = 5 each). When blood clots were continuously irradiated with a pulsed laser, the tip of the optical fiber was scorched due to coagulation of blood cell components. The optical fiber was inserted in a polyethylene tube (inner diameter: 0.40 mm, outer diameter: 0.80mm, KN-392-SP 28; Natsume Seisakusho, Tokyo, Japan), which resembled a catheter, and heparinized saline (concentration: 20 U/mL, flow rate: 6 mL/h) was continuously administered during the irradiation to prevent scorching of the blood clots. After irradiation, saline containing debris was collected by a pipette, and images of debris were taken using a phase-contrast microscope (ECLIPS E200, Nikon, Tokyo, Japan) without using Giemsa staining. The open-source image-processing software ImageJ was used to evaluate the particle size.

### Statistical analysis

An independent-samples t-test was used to determine significant differences in the data. IBM SPSS Statistics version 26 (IBM, Armonk, NY, USA) was used.

## Results

### Dynamic analysis of laser irradiation

**Fig 3:**
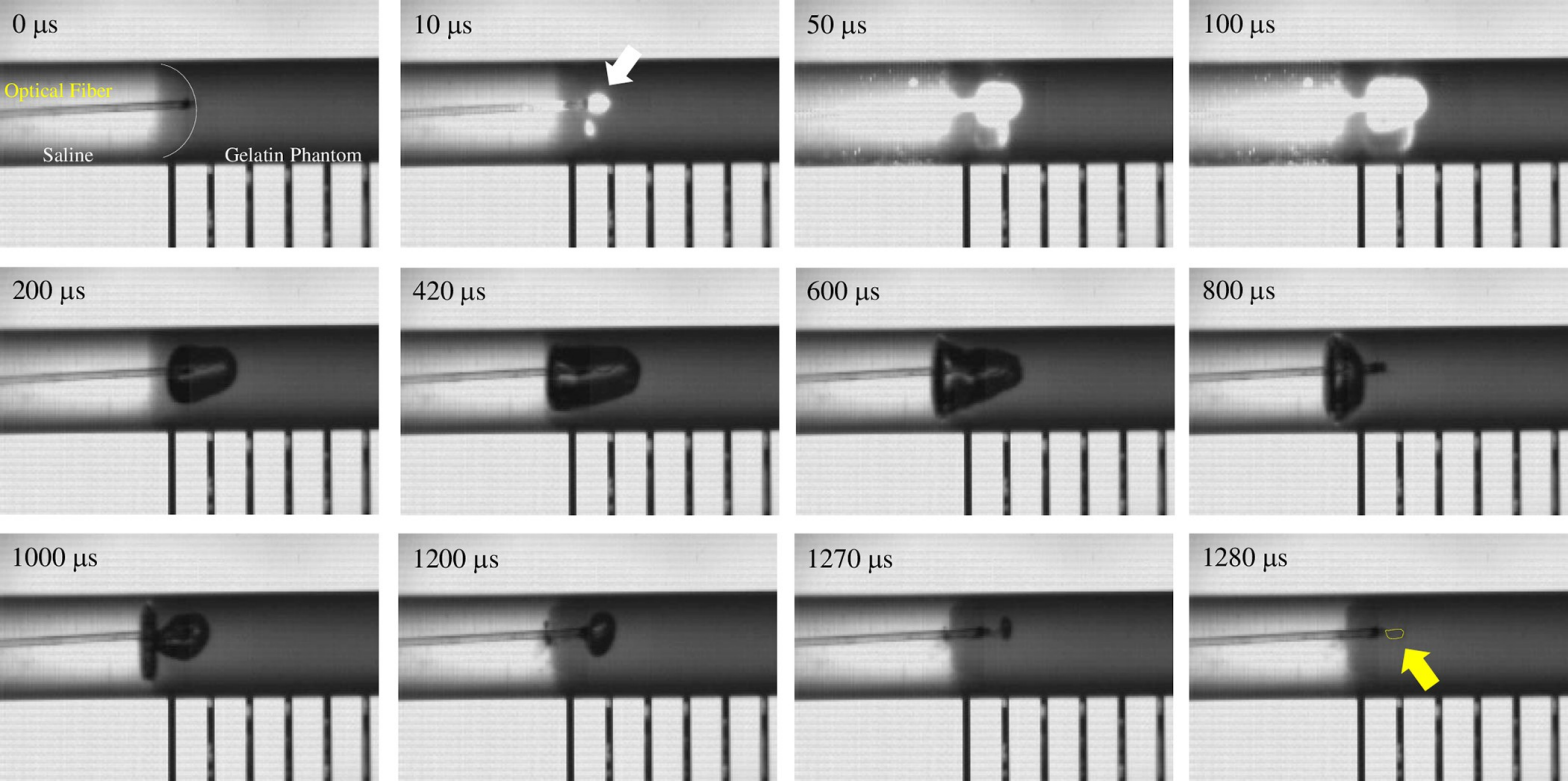
Typical example of a bubble generated by one pulsed laser irradiation in a gelatin phantom (incidence energy: 16 mJ/pulse, pulse width: 100 μs, frame rate: 10 μs/f, exposure time: 2 μs). A bubble (white arrow) in the gelatin phantom was generated within 10 μs after the irradiation onset and grew over time. The inner wall of the growing bubble was irradiated with pulsed light for 100 µs. The pulsed light was scattered and reflected within the bubble. The bubbles continued to grow even after the pulsed light irradiation ended, reaching a maximum size (long-axis direction: 2.53 mm, short-axis direction: 1.35 mm) after 420 µs. At 1280 μs, the bubble disappeared and cutting marks were observed in the gelatin (yellow arrow). No bubble bursts were observed.

**Fig 4:**
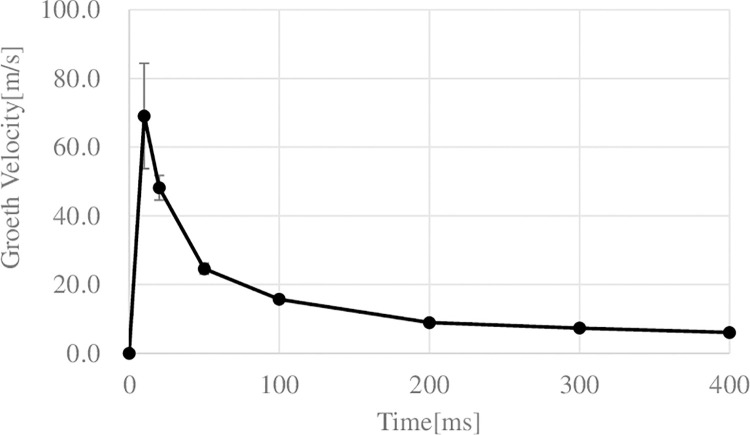
Growth velocity of bubbles generated by laser irradiation in gelatin phantom. Horizontal axis: Elapsed time after pulse laser irradiation. Vertical axis: Growth velocity of bubble in the long axis direction. The bubble growth velocity reached a maximum of 69.1 ± 15.3 m/s (N = 5) at 10 µs after the start of irradiation.

Fig 3 shows a typical reaction to one pulse laser irradiation (irradiation energy: 16 mJ/pulse, pulse width: 100 μs) in a gelatin phantom. When the laser pulse irradiated the gelatin phantom, a bubble formed at the tip of the optical fiber (Fig 3, 10 μs). The bubble (indicated by white arrow in Fig 3) was generated within 10 μs after the start of irradiation, and the rest of the pulse light irradiated the inside of the growing bubble. The generated bubbles infiltrated into the gelatin phantom and an ellipsoidal shape growing in the irradiation direction was formed. The bubbles reached a maximum size of 2.53 mm in the long-axis direction (average 2.64 ± 0.19 mm, N = 5) at 420 μs after the start of the irradiation. After that, the bubbles did not burst but shrank and disappeared, and cutting marks on the gelatin phantom were found around the tip of the optical fiber at 1280 μs after the irradiation onset. The maximum size in the short-axis direction was 1.35 mm (average: 1.42 ± 0.10 mm). After the bubble had disappeared by pulsed laser irradiation and cutting marks were observed in the gelatin phantom, the bubbles reappeared several times due to the elasticity of the gelatin phantom. This bubble generation, expansion, shrinkage, disappearance, and reappearance completely stopped after about 3300 μs (see S1 Movie). The growth rate of the bubble showed a maximum value of 69.1 ± 15.3 m/s (N = 5) at 10μs after the irradiation onset (Fig 4).

**Fig 5:**
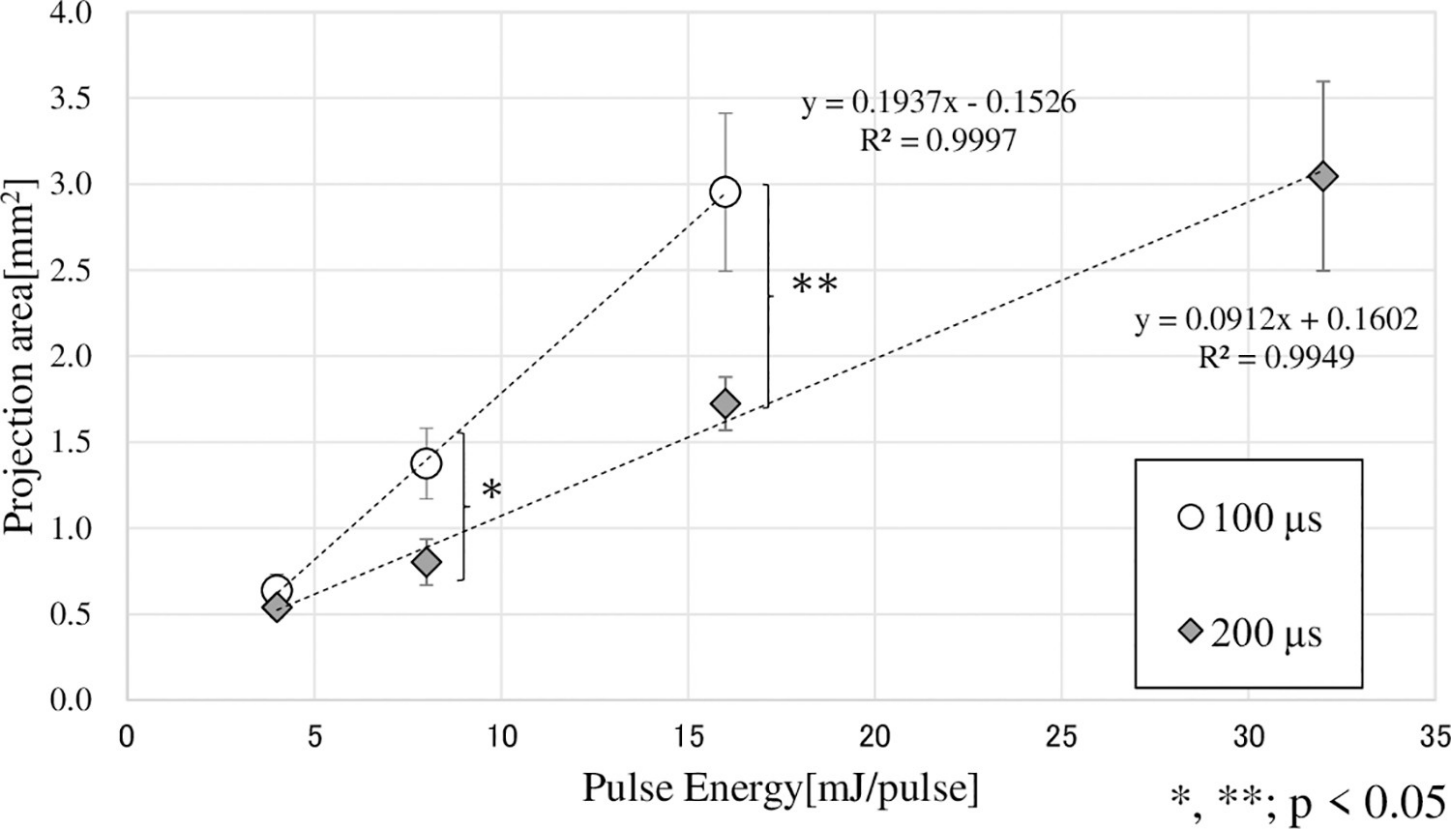
Relationship between irradiation energy and bubble projection area. ○: pulse width = 100 μs (N = 5), ♦: pulse width = 200 μs (N = 5). Horizontal axis: incident energy per pulse. Vertical axis: projected area of the bubble assuming it to be an ellipse. At both 8, 16 mJ/pulse, the bubbles that were generated with a pulse width of 200 μs were significantly smaller than those generated with a pulse width of 100 μs (*, **; p < 0.05).

Fig 5 shows the relationship between the amount of incident energy and the bubble size (projected area). As the amount of incident energy increased, the size of the bubbles also increased. However, for the same amount of incident energy, the bubbles generated with a pulse width of 200 μs for both 8 mJ/pulse and 16 mJ/pulse were significantly smaller than those generated with a pulse width of 100 μs (*, **; p < 0.05).

### Evaluating the size of debris

**Fig 6:**
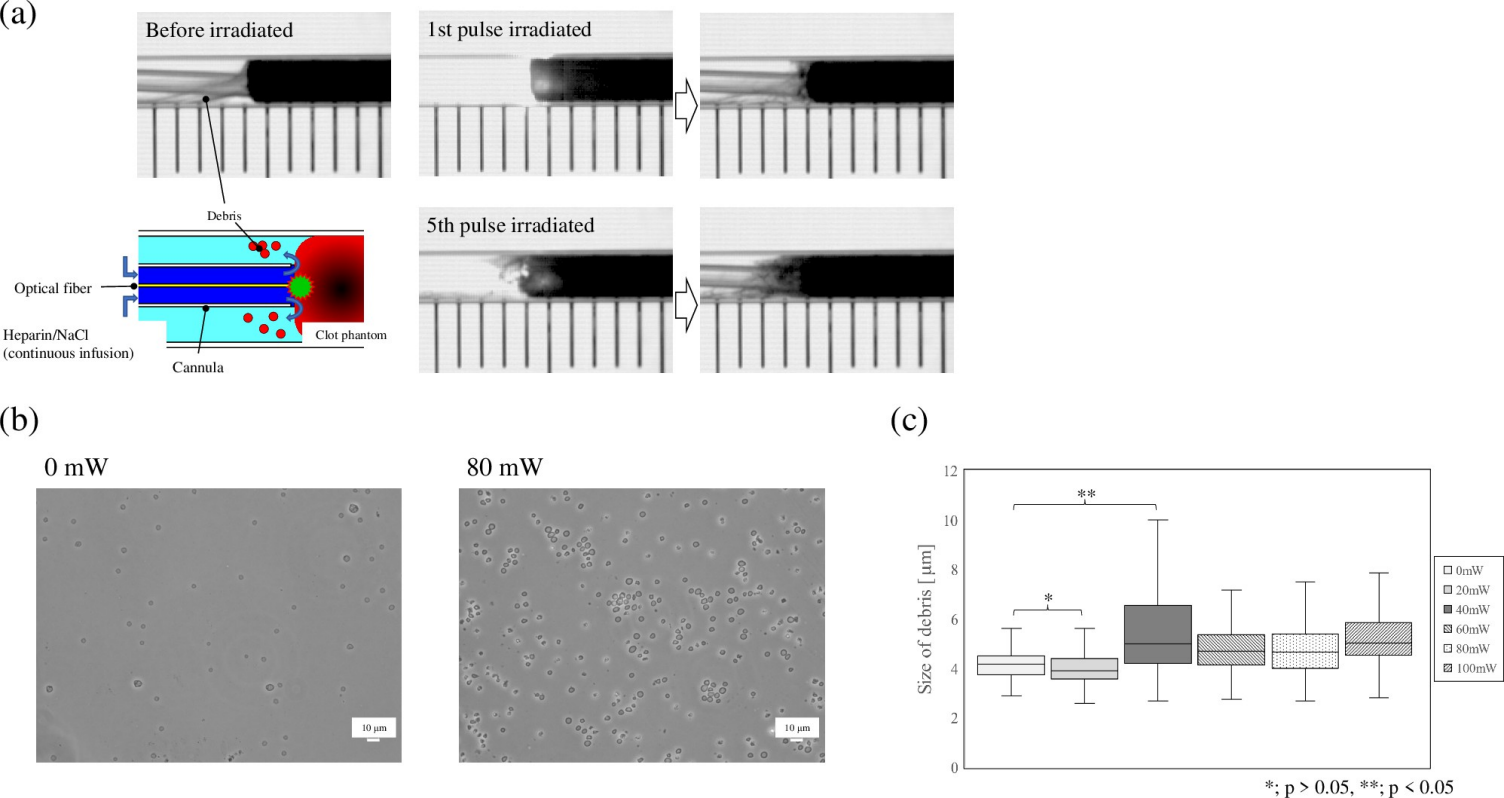
Typical response of the clot phantom to pulsed laser irradiation. (a) Block diagram and real images of the in vitro clot phantom experiment. Significant generation of debris was observed after pulsed irradiation. Incidence energy: 16 mJ/pulse, pulse width: 100 μs, frequency: 5Hz, frame rate: 60fps, exposure time: 250 μs. A glass tube (inner diameter: 2 mm) was filled with a blood clot phantom and saline solution, and pulsed laser irradiation was performed continuously for 10 seconds (total: 50 shots). The optical fiber was inserted in a polyethylene tube (inner diameter: 0.40 mm, outer diameter: 0.80mm), which resembled a catheter, to prevent scorching of the blood clots, and heparinized saline (concentration: 20 U/mL, flow rate: 6 mL/h) was continuously administered during the irradiation to prevent scorching of the blood clots. Upper group of photos: before irradiation and after irradiation by one pulse. Lower group of photos: after irradiation by five pulses. The boundary between the blood clot phantom and the saline solution became unclear as the number of irradiations increased. (b) Debris collected after 10 s of irradiation and observed under a phase-contrast microscope (without staining). The average power was 0 mW (left) and 80 mW (right). (c) Size of debris (*; p > 0.05, **; p < 0.05, Vertical axis: size of debris observed after irradiation).

The blood clot phantom was irradiated with a pulsed laser, and the size of the generated debris was evaluated. Debris leakage from the clot phantom was observed even before pulsed laser irradiation because of the effects of heparinized saline being continuously administered. However, significant debris generation was observed only after laser irradiation (Fig 6(A) and S2 Movie). Fig 6(B) shows a typical example of debris. [9] Debris consisted mainly of single red blood cells and aggregates of red blood cells. [18] The size of the largest piece of debris detected was 44 μm, but the sizes of more than 98% of the pieces of debris were less than 20 μm. There was little change in the morphology of the red blood cells. Fig 6(C) and Table 2 shows the average ratio of debris observed under each condition of average power from 0 to 80 mW (0 mW: N = 3, 20–80 mW: N = 5). More than 93% of the debris sizes were smaller than 6 μm in irradiation conditions with an average power of no more than 20 mW. On the other hand, the number of red blood cell aggregates larger than 6 μm was significantly higher at an average power of 40 mW or more (p < 0.05).

**Table 2:** Ratio of aggregated pieces generated under each irradiation condition.

	Average Power					
	0 mW	20 mW	40 mW	60 mW	80 mW	100 mW
**Less than 6 μm**	96.1%	93.5%	70.1%	85.7%	82.5%	77.3%
**6–10 μm**	3.0%	6.1%	17.8%	13.4%	9.2%	14.2%
**10–20 μm**	0.4%	0.4%	10.3%	0.9%	7.3%	7.4%
**20–50 μm**	0.0%	0.0%	1.8%	0.0%	0.9%	1.1%
**50–100 μm**	0.0%	0.0%	0.0%	0.0%	0.0%	0.0%

Results of irradiation of rabbit blood clot phantom. Pulse width: 100 μs. Repetition: 5 Hz. Debris less than 6 μm was considered to be single red blood cells. The percentage of aggregated red blood cells larger than 6 μm was recorded for each size.

## Discussion

Laser thrombolysis during laser pulse irradiation has been suggested to involve cutting by the generated bubbles and the generation of water jets as well as laser ablation, [16] and the dynamic analysis in our study showed that bubbles formed after laser irradiation. Bubbles were generated within 10 μs after the onset of pulsed laser irradiation (Fig 3). The pulse width was 100 μs, and 1.6 mJ (10% of the total incident energy of 16 mJ) was estimated to have contributed to the generation of bubbles. The absorbance A [log10] of blood (hemoglobin 4 hem, HbO_2_: 43,876 [cm^−1^/M], Hb: 40,584 [cm^−1^/M]) at 532 nm for one cell is 0.93. Assuming that 0.1 mm from the tip of the optical fiber is filled with blood, the absorbance is *A*′ = 93.0×0.01≅1, and there is no scattering in the gelatin phantom, the pulsed laser irradiation volume V is defined as a frustum of a cone in accordance with the NA of the optical fiber. Since the concentration of the gelatin phantom is 10 wt%, which can be assumed to be almost water, the amount of heat required to raise an amount of water corresponding to the volume of the irradiation area from 25℃ to the boiling point (100℃) is approximately 0.25 mJ. After the bubbles had formed, the pulsed laser irradiated the inside of the growing bubbles. Since the thermal containment time in gelatin phantom is estimated to be 35 ms [16], it was presumed the pulses between 10 and 100 μs long also had a thermal effect.

However, no bubbles were generated between 10 to 100 μs after the first bubble was generated. In the case of pulse laser irradiation with the same incident energy, the bubbles generated under irradiation conditions with a pulse width of 100 µs were larger than the bubbles generated under irradiation conditions with a pulse width of 200 μs (Fig 5). That is, the peak power of the pulsed laser affected the generation and growth of bubbles. We used an optical fiber with a core diameter of 100 μm. When a single laser pulse irradiated with an average power 80 mW (pulse width: 100 μs), we can calculate that the power density at the tip of the fiber was 2.1 MW/cm^2^. At 1 mm from the tip of the fiber, the spot size was about 0.5 mm (fiber NA: 0.22) and the power density was 81.5W/cm^2^. Moreover, the power density at an average power 20 mW was 4.1 W/cm^2^, suggesting that ablation occurred in the irradiated area of the gelatin and blood clot phantom due to the small spot size of the optical fiber irradiation [15]. At the same time, Viator et al. reported that the ablation effect was limited [16]. In this study, we used glass tubes and transparent gelatin phantoms to perform a dynamic analysis of pulsed laser irradiation by using detailed transmission images. We observed the dynamics of bubbles and the removal effect of bubbles on the thrombus immediately after laser irradiation. We found that the bubbles contracted and disappeared without bursting. The observed bubbles all formed elliptical shapes that grew in the irradiation direction. The growth velocity of the bubbles was 69.1 ± 15.3 m/s (N = 5), which was much smaller than the velocity of sound in water. T. Hirano et al. reported thrombus removal by the water-hammer effect of the laser-induced liquid jet without the generation of shock waves [19, 20]. In our experiment, the distance between the optical fiber tip and the phantom surface was set within 0.5 mm. Therefore, depending on the irradiation conditions, water may have entered between the optical fiber tip and the surface of the phantom, and may have been pushed by bubbles.

However, the sheath structure is important for the generation and control of water jets. In our study, although a certain directionality was observed in the growth direction of the bubbles, the majority of the bubbles grew in the form of infiltration into the phantom, and the effect of the water jet, which may or may not have occurred, was considered to be limited. It has also been reported that the repetitive mechanical action of water jets or an ultrasound-induced cavitation mechanism can cause fragmentation of the thrombus and increase the contact area of a fibrinolytic agent [21]. As shown in Fig 4, bubbles were generated even under irradiation conditions with an average power of 20 mW. And more than 93% of the debris was single red blood cells. Correspondingly, the percentage of erythrocyte aggregates in the debris was significantly increased under irradiation conditions with an average power of 40 mW or higher. The amount of incident energy correlates with the size of the bubbles generated, and our experimental results suggested that the deformation that the thrombus undergoes due to the generation of bubbles in an important factor in the thrombus removal effect. The blood clot phantom used in this study was coagulated whole blood with fibrin clot, which may partially reflect the structure of red blood clots targeted by laser thrombolysis therapy [22, 23]. As shown in Fig 6 (B), the phase contrast microscopy images of the debris generated by the laser irradiation showed that the red blood cells existed as discrete single cells or aggregates. In addition, there was little change in the morphology of the red blood cells. That is to say, the generation of debris, which were aggregates of red blood cells, indicates that the deformation of the thrombus phantom by air bubbles cuts the fragile part of the fibrin net. Fibrin nets are known to be fibrin glues used in surgery and other applications and have been reported to have a certain mechanical strength [24]. Although ablation occurred even at an average power of 20 mW, the result that erythrocyte aggregates did not increase significantly unless the average power was 40 mW or higher may well indicate the existence of a threshold for physical action (Fig 6 (C)). Naturally, it is thought that when bubbles are generated in a blood vessel rather than a glass tube, the vessel wall may be deformed by the pressure of the bubble generation. The bubbles did not reach the walls of the glass tube in this study, so the effect of the glass material hardness is expected to have been small [9, 17]. The glass-tube phantom model does not completely recreate an in vivo vascular situation. The absolute value of the actual duration of the bubble in vivo is thought to be variable. Similarly, it is very difficult to obtain a correlation between the mechanical strength of the phantom and the thrombus [11]. This is because the mechanical properties of thrombi formed in vivo are affected by various factors such as the passage of time and individual differences. Thus, in this paper, the mechanism of laser thrombolysis has been elucidated by using glass tubes and clear gelatin phantoms to obtain detailed transmission images, but the amount of cutting has not been examined. These problems need to be solved by constructing more detailed in vitro models in the future.

We consider that the debris generated by the laser irradiation will be released into the bloodstream and become smaller aggregates or single red blood cells through the fibrinolytic effect in vivo, similarly to what happens to small fragments generated by thrombolytic agents that act enzymatically on fibrin nets. Furthermore, we found that the bubbles infiltrate into the thrombus. This suggests that the bubbles could be used in combination with a thrombolytic agent that acts on the surface of the thrombus to enhance their effects mutually on a larger volume of thrombus.

## Conclusion

In order to elucidate the detailed mechanism of laser thrombolysis, a detailed dynamic analysis of bubbles generated by pulsed laser irradiation was carried out using transparent gelatin phantoms and clot phantoms. Our results showed that in laser thrombolysis, the thrombus could be removed by cutting by the bubbles generated by laser irradiation. In the future, we will analyze the mechanism of the reaction in more detail, including evaluating the amount of cutting, in order to determine optimal and safe irradiation conditions. In addition, we will further investigate laser thrombolysis by conducing in vivo experiments to obtain the optimal conditions for irradiation necessary for treatment and will research and develop peripheral technologies.

## Supporting information

S1 Movie Typical example of a bubble generated by one pulsed laser irradiation in a gelatin phantom. Incidence energy: 16 mJ/pulse, pulse width: 100 μs, frame rate: 10 μs/f, exposure time: 2 μs. A glass tube (inner diameter 2 mm) was filled with a gelatin phantom and saline solution, and pulsed laser irradiation was performed once using an optical fiber strand. 48914f (0 μs): Irradiation started. 48915f (10 μs after irradiation): The bubble was observed in the gelatin phantom. 48925f (100 μs): The bubble grew into an elliptical shape in the direction of the pulsed laser irradiation, and the inner wall of the growing the bubble was irradiated by the pulsed laser light. 48956f (420 μs): The bubble grew to their maximum size (The major axis: 2.53mm, the minor axis: 1.35 mm). After that, the bubble started to shrink. 49042f (1280 μs): The bubble disappeared. No bubble burst was observed. 49047f (1330 μs): The bubble reappeared due to the elasticity of gelatin. A total of four bubble reappearances and disappearances were observed since then. 49277f (3300 μs): The reaction of a one pulsed laser irradiation ended.
(AVI)

S2 Movie Typical response of the clot phantom to pulsed laser irradiation. Incidence energy: 16 mJ/pulse, pulse width: 100 μs, frequency: 5 Hz, frame rate: 60 fps, exposure time: 250 μs. A glass tube (inner diameter: 2 mm) was filled with a blood clot phantom and saline solution, and pulsed laser irradiation was performed continuously for 10 seconds (total: 50 shots). The optical fiber was inserted into a polyethylene tube (inner diameter: 0.40 mm, outer diameter: 0.80mm), which resembled a catheter, to prevent scorching of the blood clots, and heparinized saline (20 U/mL, 6 mL/h) was continuously administered during the irradiation. Pre-irradiation: Red blood cells on the surface of the blood clot phantom were observed to be discharged by continuous administration of heparinized saline. From the first shot, debris was flowed from the surface of the blood clot phantom into the saline. Thereafter, the boundary between the blood clot phantom and the saline solution became blurred as the number of irradiations increased.
(AVI)

S1 Dataset Data on which Figs 3–6C, and Table 2 were based. (XLSX)
